# Age-Related Differences of Maximum Phonation Time in Patients after Cardiac Surgery

**DOI:** 10.3390/diseases6010001

**Published:** 2017-12-21

**Authors:** Kazuhiro P. Izawa, Yusuke Kasahara, Koji Hiraki, Yasuyuki Hirano, Satoshi Watanabe

**Affiliations:** 1 Department of International Health, Graduate School of Health Sciences, Kobe University, 7-10-2 Tomogaoka, Suna-ku, Kobe 654-0142, Japan; izawapk@ga2.so-net.ne.jp; 2 Department of Rehabilitation Medicine, St. Marianna University School of Medicine Yokohama-city Seibu Hospital, 1197-1, Yasashi-cho, Asahi-ku, Yokohama 241-0811, Japan; kasahara.y@marianna-u.ac.jp; 3 Department of Rehabilitation Medicine, St. Marianna University School of Medicine Hospital, 2-16-1 Sugao, Miyamae-ku, Kawasaki 216-8511, Japan; hiraki7@marianna-u.ac.jp; 4 Department of Physical Therapy, Tokushima Bunri University, 180, Nishihama, Yamashiro-cho, Tokushima 770-8514, Japan; hirano@tks.bunri-u.ac.jp

**Keywords:** age-related difference, cardiac rehabilitation, maximum phonation time

## Abstract

**Background and aims:** Maximum phonation time (MPT), which is related to respiratory function, is widely used to evaluate maximum vocal capabilities, because its use is non-invasive, quick, and inexpensive. We aimed to examine differences in MPT by age, following recovery phase II cardiac rehabilitation (CR). **Methods:** This longitudinal observational study assessed 50 consecutive cardiac patients who were divided into the middle-aged group (<65 years, *n* = 29) and older-aged group (≥65 years, *n* = 21). MPTs were measured at 1 and 3 months after cardiac surgery, and were compared. **Results:** The duration of MPT increased more significantly from month 1 to month 3 in the middle-aged group (19.2 ± 7.8 to 27.1 ± 11.6 s, *p* < 0.001) than in the older-aged group (12.6 ± 3.5 to 17.9 ± 6.0 s, *p* < 0.001). However, no statistically significant difference occurred in the % change of MPT from 1 month to 3 months after cardiac surgery between the middle-aged group and older-aged group, respectively (41.1% vs. 42.1%). In addition, there were no significant interactions of MPT in the two groups for 1 versus 3 months (F = 1.65, *p* = 0.20). **Conclusion:** Following phase II, CR improved MPT for all cardiac surgery patients.

## 1. Introduction

Data from 2015 on life expectancy at birth show the average life expectancy of a Japanese newborn to be 80.79 years for men, and 87.05 years for women, the highest ever recorded [1]. As in other countries, the number of elderly cardiac patients in Japan is also increasing, and is becoming a growing public health problem [2,3].

Physical status has been reported in cardiac patients after the onset of acute myocardial infarction (AMI) and cardiac surgery, such as coronary artery bypass grafting and valve replacement [4,5,6,7]. The reported goals of cardiac rehabilitation (CR) programs for these patients have been to improve exercise capacity, reduce coronary risk factors, improve health-related quality of life (HRQOL), and reduce subsequent cardiac events, hospitalization costs, sudden death, and all-cause mortality [4,5,6,7].

Maximum phonation time (MPT), which is related to respiratory function, is widely used to evaluate maximum vocal capabilities, because it is non-invasive, quick, and inexpensive [8]. A previous study showed a positive correlation between MPT and poor outcome in relation to indices of exercise capacity, such as peak oxygen uptake in outpatients with chronic heart failure [9].

With regard to age-related differences in physiological outcomes, such as peak oxygen uptake and muscle strength, a previous report investigated age-related differences in exercise capacity and muscle strength [10], but the relation of age-related differences in regard to clinical characteristics and MPT in post-surgery cardiac patients is unknown. We believe that differences in age affect MPT in post-surgery cardiac patients, and thus, we hypothesized that MPT might be related to age in post-surgery cardiac patients following phase II CR. However, there is little evidence on the effects of differences in age on MPT in cardiac surgery patients. Therefore, the purposes of the present study were to evaluate (1) differences in MPT values as related to age at 1 month and 3 months after cardiac surgery, and (2) to determine the longitudinal change of MPT in relation to age in post-surgery cardiac patients following phase II CR.

## 2. Methods

### 2.1. Participants

This longitudinal study comprised 62 consecutive patients with cardiac surgery, such as coronary artery bypass grafting or valve replacement who visited a hospital as outpatients 1 month after surgery, and were referred to the department of rehabilitation medicine for first-time evaluation of MPT. Those patients with neurological, peripheral vascular, orthopedic, pulmonary, and advanced renal disease, and those on dialysis, were excluded. Patient characteristics were evaluated by review of medical records, and included age, sex, body mass index (BMI), left ventricular ejection fraction (LVEF), and etiology. We evaluated these characteristics in the patients 1 month after cardiac surgery. Patients were divided into the middle-aged group (<65 years, *n* = 35) and older-aged group (≥65 years, *n* = 27). MPT was measured at 1 and 3 months after cardiac surgery and was compared by group and term (1 and 3 months), respectively. The present study complied with the principles of the Declaration of Helsinki regarding investigations in humans, and was approved by the local institutional review board of our university (No. 612). Informed consent was obtained from each patient.

### 2.2. MPT

MPT was measured using a stopwatch (ALBA, PICCO MULTI TIMER, Seiko., Co. Ltd., Tokyo, Japan) with the patient in the seated position based on methods reported in previous studies [8,9]. Patients were asked to produce a sustained vowel/a:/sound for as long as possible, and were verbally encouraged during this effort. The method, variability, and reliability of this measurement were described previously [9]. All trials were assessed by a physical therapist. Three consecutive trials were allowed, with a 15 s break between each trial. The highest value measured was considered the index of MPT(s) [9].

### 2.3. CR Program

The supervised recovery phase II CR outpatient program continued until 3 months after surgery [4,5]. The patients participated in supervised combined aerobic and resistance exercise once or twice a week for 1 h. Each exercise session was composed of a warm-up, aerobic exercise, resistance training, and cooldown period. Exercise intensity during aerobic exercise was maintained at anaerobic threshold heart-rate level, using treadmill walking or cycle ergometer.

For resistance training, three to five sets of a series of two upper-limb exercises were performed with an iron weight array and/or handgrip at a resistance that allowed completion of five repetitions with a rating of perceived exertion of 11–13 (according to the Borg 6–20 scale). Three to five sets of a series of knee extensions, flexions, and calf raises comprised the lower-limb exercises. Knee extension was performed with a weight strapped to the ankle and at a resistance that allowed completion of five repetitions with a 40–60% of one repetition maximum. Exercise intensity for calf raises was maintained at a perceived exertion rating of 11–13. Each session was preceded and followed by series of upper- and lower-limb and body stretches [9,10].

### 2.4. Statistical Analysis

Results are expressed as mean ± standard deviation (S.D.). The unpaired *t*-test and χ^2^ test were used to test for differences between the two groups in clinical characteristics and MPT values, delta MPT values (3 months–1 month), and % change in MPT values (3 months–1 month/1 month × 100) at 1 month and 3 months after cardiac surgery. In addition, we analyzed differences in age group (middle-age group and older-age group) versus term (1 month and 3 months) using two-way repeated measures (age group versus term) ANOVA. A *p* value of <0.05 was considered to indicate statistical significance. Statistical analyses were performed with IBM SPSS 22.0 J statistical software (IBM SPSS Japan, Inc., Tokyo, Japan).

## 3. Results

### 3.1. Clinical Characteristics of the Patients

**Figure 1:**
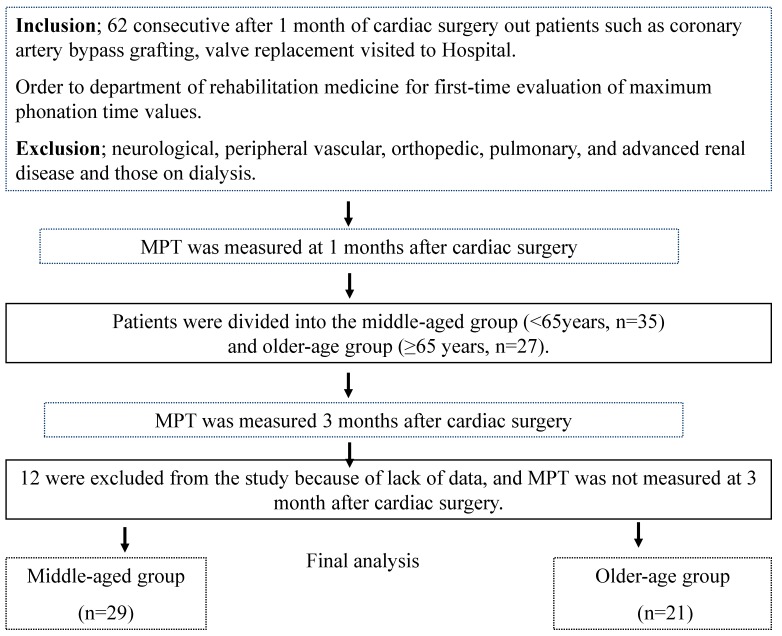
Diagram of the patient selection process.

Of the 62 patients, 12 were excluded from the study because of lack of data, or MPT was not measured at 3 months after cardiac surgery. Thus, 50 patients were divided into two groups by age: middle-aged group (<65 years, *n* = 29) and older-aged group (≥65 years, *n* = 21) (Figure 1). Except for age, patient characteristics were similar between the two groups. Clinical characteristics of the patients are presented in Table 1.

### 3.2. Age-Related Differences in MPT at 1 Month and 3 Months

MPT data collected from the two groups are presented in Table 2. MPT values in the older-aged group were significantly lower than those in the middle-aged group at both 1 month and 3 months after surgery.

### 3.3. Effects of Aging Following Phase II CR

After phase II CR outpatient programs, both the middle-aged group and older-aged group showed statistically significant improvements, in both delta and % change of MPT from 1 month to 3 months after cardiac surgery (Table 2). No statistically significant difference occurred in the delta and % change of MPT from 1 month to 3 months after cardiac surgery between the middle-aged group and older-aged group, respectively (Table 2). In addition, no significant period (from 1 month to 3 months) by group interactions (middle-aged and older-aged groups) (MPT: *F* [1/48] 1.65, *p* = 0.20) was detected. No patient showed ischemic ST changes or experienced chest pain or serious arrhythmia during the phase II CR outpatient program.

## 4. Discussion

This is first time, to our knowledge, that the MPT of cardiac surgery patients undergoing a phase II CR outpatient program has been evaluated in relation to age. The main findings of this study are that in measurements of MPT, the older-aged group had lower MPT than the middle-aged group at 1 month and 3 months after cardiac surgery. However, the increase in MPT from 1 month to 3 months in the older-aged group was similar to that in the middle-aged group.

Except for age in the present study, other patient clinical characteristics were almost identical between the two groups. Therefore, clinical characteristics other than age might not affect the differences of MPT observed between the two groups. Significant differences were present in MPT measures at 1 month and 3 months after cardiac surgery, in relation to age, indicating that aging might reflect reduced MPT in elderly cardiac surgery patients. Previously, Izawa et al. [9] evaluated MPT in relation to exercise capacity in chronic heart failure outpatients, and found that a low exercise capacity level was associated with decreased MPT following phase II CR.

Exercise capacity is very important in relation to mortality, morbidity, HRQOL, and activities of daily living in cardiac patients [4,5,6,7]. A previous study reported that baseline exercise capacity (estimated METS) was lower in elderly patients (≥65 years old) than in younger patients (<65 years old) [11]. Another study also suggested that peak oxygen uptake during exercise in the older-aged group was significantly lower than that in the middle-aged group at 1 month after the onset of AMI and after cardiac surgery [10]. Even though the etiology of the present-study patients was different from that of the patients with chronic heart failure and AMI, the present study showed that the MPT of older cardiac surgery outpatients, at risk for poor exercise capacity, may be affected, and supports the findings of these previous studies.

Although phase II CR outpatient programs have been shown to have benefits after AMI and cardiac surgery, many previous reports have focused on an age bias in the approach to treating elderly patients. In the present study, there were significant improvements in MPT from 1 to 3 months in both groups (Table 2). We previously reported that phase II CR outpatient programs for cardiac patients after AMI, coronary artery bypass grafting, and valve replacement improved physiologic outcomes similar to those found in the present study [4,5,10]. Another study also indicated that improvement of MPT was similar to that of exercise capacity in chronic heart failure patients following phase II CR [12]. These findings may support our present study results.

However, there was a difference in the recovery process between the groups in one of these previous studies [10] in regard to peak oxygen uptake and knee extensor muscle strength in cardiac patients after AMI, coronary artery bypass grafting, and valve replacement, both of which showed greater improvement in the middle-aged group than in the older-aged group. Although the different etiology of these previous patients, which included AMI, precludes direct comparison of the previous study results with those of the present study, the improvement in MPT may be similar between the groups of these two studies.

With regard to MPT in community-dwelling Japanese people (380 adults [246 women, 134 men; age, 72.7 ± 5.9 years]), Shinoda et al. previously reported that average MPT values of age groups 60–69, 70–79, and 80–89 years, were 17.9, 16.8, and 15.9 s, respectively [13]. In the present study, MPT values in the older-aged group at 1 month and 3 months were 12.6 s and 17.9 s, respectively, which were lower, but still similar to those of the community-dwelling Japanese people. Therefore, we need to evaluate whether MPT continues to change over the long term following a phase II CR outpatient program.

Limitations in the present study include its very small sample size that comprised only a few female cardiac surgery patients. Additional analysis of sex-related differences in MPT in relation to age in cardiac female patients is needed. The effects of medications, renal dysfunction, anemia, smoking, and incubation time or period were not investigated in this study, and will require investigation in future studies. We also did not evaluate the effects of postoperative complications or the starting day of the earlier phase I CR program on selection bias pertaining to MPT testing, or of longer hospital stays on MPT. In addition, we did not evaluate the other rehabilitation programs, for example, speech therapy was not completely the same for the patients.

Data on the timing of MPT testing in relation to cardiac-related mortality or re-hospitalization were not assessed, due to the limited amount of related data available. Therefore, we need to address these deficiencies in future longitudinal studies.

## 5. Conclusions

This study identified age-related differences in MPT in Japanese cardiac surgery patients who underwent a phase II CR outpatient program. Baseline age-related differences indicated that older cardiac patients may have a lower MPT than middle-aged cardiac patients at 1 month after entrance into this type of CR program, and at 3 months after surgery. However, there were similar improvements of MPT in both the middle- and older-aged groups. Therefore, following phase II CR improved MPT for all cardiac surgery patients.

This relatively short-term study lacks long-term follow-up data, and additional study will be required to evaluate whether such CR outpatient programs can influence either long-term outcomes and age-related differences in MPT over longer periods in these patients, or increases in other physiological outcomes of cardiac surgery patients.

## Figures and Tables

**Table 1:** Clinical characteristics of the patients.

Group	Middle-Aged Group	Older-Aged Group	*t* or χ^2^ Value	*p* Value
No. of patients	29	21		
Age (years)	56.3 ± 7.6	71.1 ± 3.6	−8.15	<0.001
Sex (male)	26	17	0.76 *	0.31
BMI (kg/m^2^)	24.3 ± 7.7	23.1 ± 2.6	0.71	0.47
LVEF (%)	51.6 ± 13.1	55.3 ± 12.7	−1.01	0.32
Etiology (%)				
CABG	18	16	0.29 *	0.36
VR	11	5	-	-

BMI body mass index; LVEF left ventricular ejection fraction; CABG coronary artery bypass grafting; VR valve replacement; * χ^2^ value.

**Table 2:** MPT from 1 month to 3 months.

MPT	Middle-Aged Group	Older-Aged Group	*t* Value	*p* Value
1 month (s)	19.2 ± 7.8	12.6 ± 3.5	3.63	0.001
3 months (s)	27.1 ± 11.6	17.9 ± 6.0	3.29	0.001
Delta (3 months–1 month)	7.8 ± 7.9	5.3 ± 5.0	1.29	0.21
% Change (3 months–1 month)/1 month × 100	41.1 ± 63.2	42.1 ± 43.4	0.18	0.85

MPT: maximum phonation time.
